# Roadmap for achieving net-zero emissions in global food systems by 2050

**DOI:** 10.1038/s41598-022-18601-1

**Published:** 2022-09-05

**Authors:** Ciniro Costa, Eva Wollenberg, Mauricio Benitez, Richard Newman, Nick Gardner, Federico Bellone

**Affiliations:** 1 The Alliance of Bioversity International and CIAT–Multifunctional Landscapes, Cali, Colombia; 2 The Alliance of Bioversity International and CIAT–Sustainable Finance, Cali, Colombia; 3 Gund Institute for Environment, University of Vermont, Burlington, VT USA; 4 Clim-Eat, Wageningen, The Netherlands; 5 responsAbility Investments AG, Zurich, Switzerland; 6 U.S. Dairy Export Council, Arlington, VA USA; 7 UNFCCC Climate Champions, Bonn, Germany

**Keywords:** Climate-change mitigation, Environmental impact, Projection and prediction

## Abstract

Food systems (FSs) emit ~ 20 GtCO_2_e/y (~ 35% of global greenhouse gas emissions). This level tends to raise given the expected increases in food demands, which may threaten global climate targets. Through a rapid assessment, evaluating 60+ scenarios based on existing low-emission and carbon sequestration practices, we estimate that intensifying FSs could reduce its emissions from 21.4 to − 2.0 GtCO_2_e/y and address increasing food demands without relying on carbon offsets (e.g., related to afforestation and reforestation programs). However, given historical trends and regional contexts, a more diverse portfolio of practices, including diet shifts and new-horizon technologies, will be needed to increase the feasibility of achieving net-zero FSs. One likely pathway consists of implementing practices that shift food production to the 30th-percentile of least emission-intensive FSs (~ 45% emissions reduction), sequester carbon at 50% of its potential (~ 5 GtCO_2_e/y) and adopt diet shifts and new-horizon technologies (~ 6 GtCO_2_e/y). For a successful transition to happen, the global FSs would, in the next decade (2020s), need to implement cost-effective mitigation practices and technologies, supported by improvements in countries’ governance and technical assistance, innovative financial mechanisms and research focused on making affordable technologies in the following two decades (2030–2050). This work provides options and a vision to guide global FSs to achieving net-zero by 2050.

## Introduction

The Paris Agreement’s goal of limiting the increase in global temperature to 1.5° above pre-industrial levels requires rapid and ambitious reductions in global greenhouse gas (GHG) emissions. This can only be achieved by drastic emissions reductions across the energy; industry; transport; buildings; and agriculture, and forestry sectors^1,2^.

Even if fossil fuel emissions stopped now, current trends in global food systems (FSs) would prevent the achievement of the 1.5 °C target and threaten the achievement of the 2 °C target by the end of the century^3^. However, carbon budgets or net-zero emissions are often only discussed for CO_2_ emissions and not for non-CO_2_ emissions, such as CH_4_ and N_2_O, in which FSs, especially agriculture production, are the main source^3–5^.

Today, FSs GHG emissions contribute to roughly a third of global emissions. In 2019, FSs emitted 16.5 (95%; CI range: 11–22) GtCO_2_e globally, the largest contributors were agriculture, land use, land-use change activities (~ 70%) and the remaining emissions coming from other downstream and upstream activities (i.e., retail, transport, consumption, fuel production, waste management, industrial processes and packaging)^6^. Since global food production is estimated to increase by 15% in coming decades^7^, FSs emissions might increase by up to 80% from 2010 to 2050^3,6,8–11^. In addition, there are still almost 700 million people undernourished and living under severe food insecurity^12^ who must be considered in FS planning. Therefore, the Paris Agreement and Sustainable Development Goals can only be achieved with significant contributions from FS, including supply-side measures in agriculture production and demand-side measures related to diet changes and reduced food waste^5,13^, while strengthening food security and safety^14^.

Substantial GHG emissions reductions in FSs are attainable by implementing low-emission interventions to improve efficiency and nature-based carbon sequestration^3,5,15^. Low-emission interventions could result in ~ 40–70% less GHG intensive production systems compared to today’s average levels^16^. Additionally, a carbon sequestration potential, of approximately 10 GtCO_2_ y^-1^, is associated with FSs production under the expansion of agroforestry systems, improved pasture and crop management and application of biochar to soils^5^.

Nevertheless, the mitigation benefits of improved systems could be offset under food production’s current growth trajectory, especially for livestock production^10^. Even with higher efficiency, greater production needed to meet growing demand might increase net GHG emissions. This condition suggests that dietary changes, including a reduction in consumption of livestock products and replacement by plant-based foods, is also important to help transition to low-carbon and net-zero food systems^5,8,13,15^. Furthermore, several technologies developed or under development might help further reduce emissions in the medium and long run, such as feed additives for livestock, novel perennials, soil additives, nanoproducts and intelligent food packaging^17^.

Therefore, a combination of actions (e.g., implementation of low-emissions interventions for improving production systems efficiency, promotion of carbon sequestration; reduction in livestock-based protein consumption and deployment of new-horizon technologies) is likely necessary to reduce net GHG emissions of FSs aligned with net-zero emissions strategies^10,15^.

Although the impressive commitment to the net-zero agenda of countries and the world’s biggest food companies, guidance offering multiple options for achieving net-zero emissions in global FSs and informing the effectiveness of pledges and catalyze meaningful climate action is still needed. To date, most studies have focused on estimating global food systems emissions^6,18^ and evaluating potential mitigation through a few and aggregated pathways using complex models^3,10^ and none has proposed a roadmap towards net-zero food systems, which has lately been highly demanded by several food systems actors^19^.

Through a rapid assessment using three datasets, the FAO forecast on global food production by 2050^7^ and food value-chain emissions intensities^16^ and carbon sequestration potentials^5^, we built 60+ pathways towards 2050 by analyzing global food demands with the implementation of four major interventions in FSs: (1) implementing low-emission practices to reduce emissions through increased production efficiency (10th, 20th, 30th, 40th pctl of least emission-intensive systems and average); (2) sequestering carbon in croplands and grasslands; (3) shifting diets to reduce global production of livestock-based protein; and (4) adopting new-horizon technologies across food value-chains. In calculating these contributions, we also provide a vision, with examples, to downscale global sectoral goals to the regional level, highlighting areas where improvements are needed. It is important to note that since our analysis is limited to a global overview, the implications of FSs intensification may have different consequences at regional scales. Further analysis is needed to shed more light on the possibility to mix different intensification strategies to optimally meet socio-economic and environmental targets. However, as countries and companies begin implementing their pledges and establish sectoral targets, our analysis provides a transparent, scientific basis for gauging the ambition of these contributions to global net-zero food systems.

## Results

### Food system emissions snapshot

**Figure 1:**
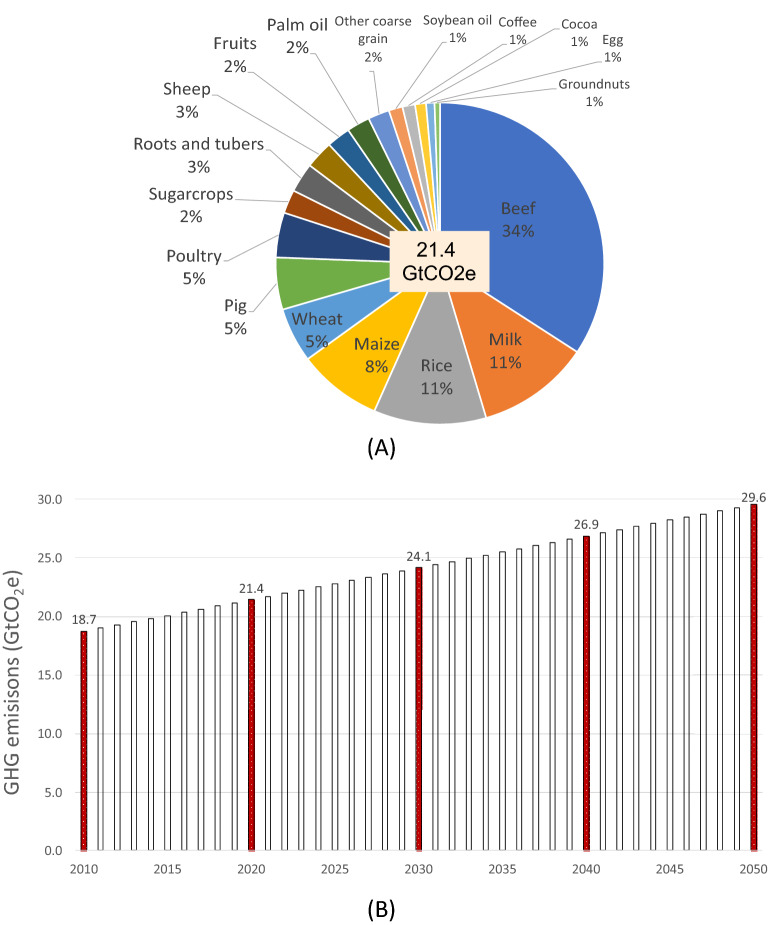
Global food systems emissions in 2020 (**A**) and estimated global food systems emissions 2010–2050 (**B**).

We estimate that global FSs emitted 18.7–21.4 Gt CO_2_e/y from 2010 to 2020 (Fig. 1). This estimate is consistent with the emissions range of recent estimates covering the same period (9–22 GtCO_2_e/y)^3,6,16,18,20–22^. Four value-chains–beef, milk, rice and maize–are responsible for nearly 65% (13.9 GtCO_2_e) of total FS emissions, and seven value-chains (+ wheat, pig and poultry) are responsible for almost 80% of emissions (17.2 GtCO_2_e). Livestock production (meat and milk) alone accounts for 60% of total FSs emissions (12.6 GtCO_2_e) (Fig. 1). Close to 70% of FS emissions come from land-use change and farming activities^6,16^.

The production of major grains, meat and milk is projected to increase 29–81% by 2050 compared to today’s levels^7^. Under current average production practices, meeting the 2050 projected food production^7^ would increase FS emissions by 38% (~ 8 GtCO_2_e/y) compared to 2020, respectively (Fig. 1). These findings are consistent with recent analyses that have suggested that global FS emissions might increase 30–50% by 2050^11^.

### Mitigation potential of low-emission and carbon sequestration food production practices

**Figure 2:**
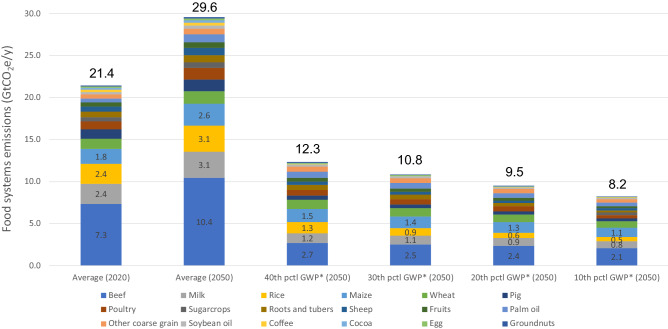
Food systems emissions by shifting global food production to the 40th, 30th, 20th and 10th pctl least emission-intensive systems in 2050.

We find that the adoption of low-emission practices could shift global FSs production from the average to the 40th, 30th, 20th and 10th-percentile (pctl) of least emission-intensive systems^16^ and could reduce the emission of 9.1–13.2 GtCO_2_e/y in 2050 compared to the 2020 base year level (21.4 GtCO_2_e) (Fig. 2). Major contributions would come from livestock and rice value-chains (Fig. 2).

Although these FS value-chains are the most emission-intensive ones, they also have the largest mitigation potential across FSs (Fig. 2). For example, improving production practices with existing technologies could reduce emissions by 40%-70% compared to average values: beef from 7.3 to ~ 2.5 GtCO_2_e/y and rice and milk from 2.4 to ~ 1.0 GtCO_2_e/y (Fig. 2). Most of this mitigation potential is related to reductions in land-use change (e.g., deforestation for agricultural land expansion), improvements in animal feeding and breeding and manure management, nutrient management (with focus on nitrogen fertilizers), water management in rice paddies and energy efficiency (e.g., renewables) across the value-chain as well as measures to reduce food loss and waste (i.e., improved packaging and storage)^5,16,17,23,24^. Also, using a global warming potential accounting for short-lived GHGs (GWP*), like CH_4_, means that relatively small annual reductions in CH_4_ emissions (~ 0.3%) could eliminate global warming caused by the emissions of CH_4_ from biogenic sources in 20 years^25–27^.

Harnessing the carbon sequestration potential associated with low-emissions agricultural practices could contribute to an additional emission abatement of 10.5 GtCO_2_e/y^5^. Most of this potential is related to the below- and above-ground carbon accumulation with the expansion of agroforestry systems (5.6 GtCO_2_e/y) and soil carbon sequestration with improvements of pasture and crop management (2.5 GtCO_2_e/y), such as the adoption of reduced and no-tillage and grass-legume mixtures in pastures, and the application of biochar to soils (2.4 GtCO_2_e/y)^5^. Furthermore, it is worth noting that these mitigation actions also have synergies with food productivity, climate adaptation, and other environmental aspects (e.g., water and soil conservation)^17,28,29^.

### Reduction in livestock-based protein consumption

Reducing livestock-based protein consumption is often pointed out as another option to reduce GHG emissions from food systems^3,8^. Nevertheless, under current average livestock production practices, a reduction of livestock-based protein consumption would only decrease livestock emissions in 2050, compared to the 2020 levels, if projected production is cut over 25% (Table 1). At or below this level, livestock emissions would rise or be kept constant considering today’s average production system emissions and projected increases in meat (+ 37%) and milk (+ 29%) productions by 2050^7^ (Table 2). On the other hand, if accompanied by the implementation of low emission practices, reducing the consumption of livestock-based protein by 10% and 25%, for example, could promote emission reductions of 0.5–2.5 GtCO_2_e/y by 2050 (Table 1). Therefore, scaling the implementation of low-emissions practices to improve livestock production is a precondition to drive significant changes in emissions towards net-zero FSs.

**Table 1:** Estimated and projected meat and milk production and emissions as a function of consumption reduction in 2020 and 2050.

Production	2020	2050	Level of consumption reduction by 2050		
	Total	Total	− 10%	− 25%	− 50%
Meat (M ton)	330.3	452.1	406.9	339.1	226.1
		Compared to 2020			
		36.9%	23.2%	2.7%	− 31.6%

			− 10%	− 25%	− 50%
Milk (M liter)	825.0	1065.0	958.5	798.8	532.5
		Compared to 2020			
		29.1%	16.2%	− 3.2%	− 35.5%

Emissions (tCO2e)					
			− 10%	− 25%	− 50%
Average	12.6	17.5	15.7	13.1	8.7
40th pctl		5.4	4.9	4.1	2.7
30th pctl		5.0	4.5	3.7	2.5
20th pctl		4.6	4.1	3.4	2.3
10th pctl		3.9	3.5	2.9	2.0
		Compared to 2020			
Average		38.6%	24.7%	3.9%	− 30.7%
40th pctl		− 56.8%	− 61.1%	− 67.6%	− 78.4%
30th pctl		− 60.4%	− 64.4%	− 70.3%	− 80.2%
20th pctl		− 63.6%	− 67.3%	− 72.7%	− 81.8%
10th pctl		− 69.0%	− 72.1%	− 76.8%	− 84.5%

Based on FAO, 2018; Poore and Nemecek^16^.

**Table 2:** Mitigation potential of food systems practices.

LED and C-sequestration practices priorities by cost	2020–2030	2030–2040	2040–2050	Mitigation potencial/cost*^5^	
				Up to 100 US$/tCO2e	> 100 US$/tCO2e
**Existing practices and technologies**				**47%**	**53%**
Rice paddies	Improved water management in rice paddies			70%	30%
Crop	Nutrient management (e.g., balance nitrogen application)			87%	13%
	Biochar			77%	23%
	No-till and residue management			90%	10%
Livestock	Grazing management; animal feeding, health and breeding and feed additives			61%	39%
	Manure management			78%	22%
Cross-cutting (crop-livestock)	Agroforestry			20%	80%
Off-farm/demand side/other	Avoided forest conversion			59%	41%
	Reduce food loss and waste			52%	48%
	Shift diet demands from livestock- to plant-based protein			63%	37%
	Renewable energy and improved fuel efficiency			–	–
**New horizon technologies**					
Rice paddies	Plant and soil microbiome technology; perennial row crops			–	–
Crop	Enteric methane direct capture, new inhibitors and novel feed additives			-	–
Livestock	Gene editing for enhanced carbon sequestration			–	–
Cross-cutting (crop-livestock)	New technologies—not yet present but could increase mitigation from GHG–efficient food production practices			–	–
Off-farm/demand side/other				–	–
Development	Affordable and available				

### New-horizon technologies

New technologies to reduce GHG emissions from FSs include those that are still costly (Roe et al.^5^) and primarily not yet present in food value-chains but could increase mitigation from GHG–efficient food production practices, land-use change, and carbon sinks^30^. This diverse pipeline, including consumer-ready artificial meat, methane inhibitors, intelligent packaging, vertical agriculture, nano-drones and 3-D printing, presents real opportunities for systemic change^17^. Also, if these technologies are developed to reduce costs of existing agricultural-related practices that are not cost-effective today (e.g., > 100 USD/tCO_2_e), it could unlock emissions reductions and carbon sequestration of approximately 8.5 GtCO_2_e/y, representing close to 40% of today’s FSs emissions and 50% of agricultural-related mitigation potential^5^. For example, the implementation of agroforestry has the technical potential to sequester approximately 11.2 GtCO2e/y, but only 20% of this potential is considered cost-effective today^5^.

### Food system mitigation potential

**Figure 3:**
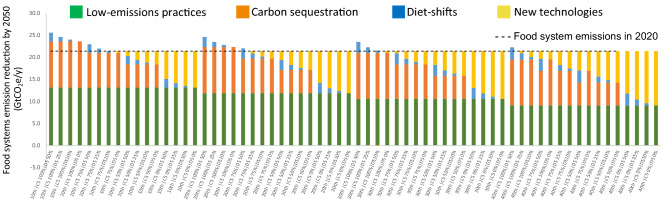
Food systems emissions reduction (green bar) with the implementation of low-emission practices (to move production systems to the least 10th, 20th, 30th and 40th pctl emissions intensive*), realization of potential carbon sequestration (CS) in agriculture in soils, agroforestry and biochar application (CS; at 0, 50, 75 and 100% level of implementation**), diet shift (DS) to reduce livestock-based protein consumption (SD; at 0, 10, 25 and 50% of projected 2050 values***) and adoption of new-horizon technologies (orange bar). *(Poore and Nemeck^16^); **(Roe et al.^5^; 10 GtCO_2_e); ***(Based on 2050 projected meat and milk projections—FAO, 2018).

By randomly combining the implementation of major FS mitigation actions to target net-zero emissions by 2050 in 64 scenarios, we found that only eight would lead to net-zero FSs through the implementation of existing low emission and carbon sequestration production practices (Fig. 3), another eight scenarios would need to further rely on diets shifts and the remaining 48 would need additional emission reduction with the implementation of new-horizon technologies reducing up to 5 GtCO_2_e/y (Fig. 3).

Through the implementation of existing low-emission and carbon sequestration practices only (i.e., excluding the reduction in livestock-based protein consumption and new-horizon technologies), we estimate that FSs emissions could shift from 21.4 to ~ − 2.0 GtCO_2_e/y by 2050 (i.e., 110% reduction compared to 2020 level by moving FS to the 10th pctl of least emission-intensive practices and harnessing 100% of the carbon sequestration potential) (Fig. 3).

The higher the implementation of low-emissions practices (i.e., towards the 10th pctl of leasts emission-intensive systems), the lower the dependance on carbon sequestration, reduction of livestock-based protein consumption and new-horizon technologies. Therefore, scaling low-emissions practices to improve FSs production is fundamental to feasibly driving significant changes in emissions towards net-zero FSs (Fig. 3).

The conditions for harnessing the full FSs mitigation potential in the next three decades are ambitious given the cost-effectiveness of practices, differences in regional contexts (e.g., cost of implementation, institutional and technical capacity, and food access and demands), historical trends and uncertainties related to carbon sequestration^5,11,13,31,32^. For example, over the last 30 years (1988–2017), global productivity of cereals, rice, beef and dairy increased 9–40% while emission intensity (at farm level—major emission source; Fig. 2) was reduced by 7–40%, respectively (FAO-Stat, 2021). These numbers are far behind the emission reduction potential of ~ 65% (i.e., 10th pctl least emission-intensive systems) and more compatible with the 40th pctl least emission-intensive systems (Fig. 2). Only about 50% of the technical mitigation potential of existing agricultural-related practices and technologies are cost-effective today (e.g., up to 100 USD/tCO_2_e), and close to 75% of that is in developing (~ 65%) and least developed (~ 10%) countries (Roe et al.^5^). This may add extra financial, technical and policy constraints for implementing FSs net-zero emissions plans, as developing and least developed nations likely have lower institutional capacity for implementing more effective climate policies^33^.

There are still concerns regarding carbon sequestration permanence, which encompasses issues related to the time and vulnerability of the carbon sequestered in soils and biomass, such as (i) differential sequestration rates over time and long run decline to a near-zero rate, and (ii) release of sequestered carbon back into the atmosphere after discontinued carbon sequestering practices^31,32,34^. These aspects suggest that bolder actions to mitigate GHG from FSs are necessary to increase chances to achieve net-zero FSs emissions by 2050; according to the strategies and assumptions evaluated in this work, there is no silver bullet, and a combination of actions should therefore be targeted to increase the feasibility of achieving net-zero emission FSs by 2050 (Fig. 3).

### The roadmap for net-zero food systems

Without relying on carbon offsets (e.g., related to afforestation and reforestation), FSs have the potential to reach net-zero emissions by 2050 (Fig. 3), but countries’ contextual constraints are likely to limit the potential reach of implementation. However, recent engagement of global FSs actors, along with advances in the plant-based protein industry and disruptive technologies^17,35,36^, has created momentum for action that may speed the implementation of low-emission and carbon sequestration practices, as well as the dissemination of diet shifts, to move FS emissions away from current trends. In this context, a vision for a net-zero FSs encompasses:

- Large-scale adoption of low-emission practices to shift the production to the 30th pctl of least emission-intensive systems (~ 45% emissions reduction across FSs), which could mitigate 10.6 GtCO_2_e/y, or ~ 50% of the mitigation needed by 2050 compared to the 2020 base year.
- Realizing 50% of the carbon sequestration potential associated with low-emission practices (i.e., soil carbon, agroforestry and biochar) could contribute another ~ 24% (5.2 GtCO_2_e/y) emission reduction.
- Reducing the remaining FS emissions (5.6 GtCO_2_e/y) by decreasing 2050 projected livestock production, especially in high- and middle-income countries, in 25% (1.2GtCO_2_e/y) and by deploying new-horizon technologies (4.4 GtCO_2_e/y) (Fig. 3).

**Figure 4:**
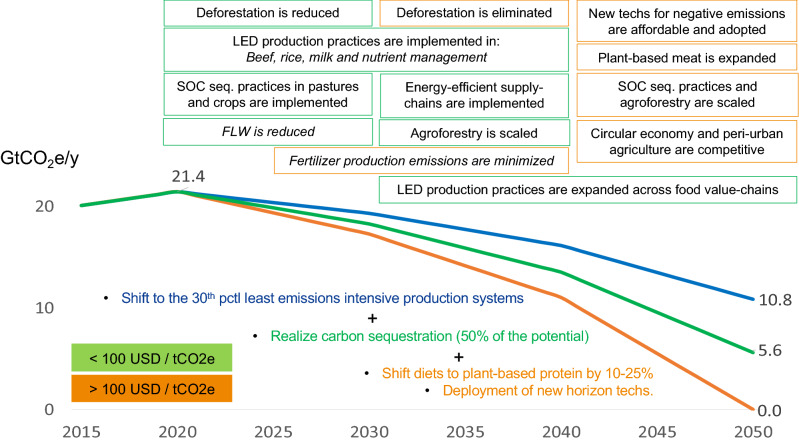
Roadmap for food systems net zero emissions by 2050.

Major actions to implement this vision over the next three decades could be summarized as follows:

- By 2030, implement cost-effective actions to reduce CO_2_ emissions from land-use change (e.g., deforestation and other land conversion) for food production along with using existing technologies to improve (i) beef, milk and rice production and (ii) nutrient management (focusing on nitrogen fertilizer) across major grain production systems (e.g., maize and wheat). By 2040, low-emissions agricultural practices should be implemented to harness the remaining cost-effective mitigation potential. Of this mitigation potential, 55% to 87% could be achieved with practices costing up to 100 US$/tCO_2_ (Fig. 4; Table 2).
- Implement cost-effective technologies and practices to sequester 1.7, 3.5 and 5.2 GtCO_2_ annually by 2030, 2040 and 2050, respectively. This can be achieved by adopting agroforestry, applying biochar to soils and improving crop (e.g., tillage and cover crops) and pasture management (e.g., rotational grazing and fertilization) practices. Close to 45% of the carbon sequestration potential (4.8 GtCO_2_ y^−1^) would cost up to 100 US$/tCO_2_ (Fig. 4; Table 2).
- By 2040, scale the use of renewable energy (e.g., wind and solar), enhance fuel efficiency, expand the electric transportation fleet, improve fertilizer production, expand the circular economy and peri-urban agriculture, and promote diet shifts in high- and middle-income countries (Fig. 4; Table 2).
- From 2040 to 2050, develop and produce affordable new-horizon technologies for negative emissions, with focus on livestock production systems (e.g., methane capture, feed additives and new breeds), novel plants and perennials for carbon sequestration and enhanced energy efficiency for storing, processing, transporting, packaging and retailing. Approximately 5.6 GtCO_2_e/y (2.6 and 5.7 GtCO_2_e emissions reduction and carbon sequestration, respectively)—or ~ 25% of the mitigation needed for net-zero FS—could be unleashed with the reduction of implementation costs (today above 100 US$/tCO_2_) (Fig. 4; Table 2).

### Making net-zero food systems realistic

Our results show that the implementation of major mitigation actions for intensifying FSs based on existing low emission and carbon sequestration practices have the potential to reduce FSs emissions beyond net-zero by 2050 while increasing food production. Our analysis also demonstrates that an intensification strategy with a more diverse portfolio of practices, most notably diet shifts and new-horizon technologies, will be more effective for reaching net-zero emissions by 2050 without relying on carbon offsets (e.g., related to afforestation and reforestation).

Even so, this scenario may not be realistic under today’s trends considering that net-zero FSs require reducing emissions by 3.3% or ~ 700 MtCO_2_e annually between 2020 and 2050. In 2020, global fossil fuel emissions dropped 5.4% as a consequence of the COVID-19 pandemic, which is an unprecedented emissions reduction (at least since 1970)^37^. However, as the global economy is rebuilt, a rebound of 4.8% is expected in 2021^37^, leaving a net emissions reduction of just 0.6%. These numbers illustrate how difficult and massive the challenge to change current production patterns and reduce emissions is.

This scenario could be different for FSs given the recent engagement of global FSs actors with the climate agenda and climate commitments (e.g., UNFSSS, Global Methane Pledge, and SBTi)^38^. Along with significant advances in the plant-based protein industry and disruptive technologies, this engagement has created a momentum for action that may speed up the implementation of steps to move FSs emissions away from business-as-usual trends.

Against this backdrop, implementing cost-effective measures and making affordable practices and new-horizon technologies in the coming decades seems to be a reasonable mitigation pathway for increasing the chances of food systems achieving net-zero emissions by 2050. To make net-zero FSs realistic it is essential to overcoming barriers, for example, related to regional contexts (e.g., cost of implementation, institutional and technical capacity, and food access and demands), historical trends, and uncertainties related to carbon sequestration^5,11,13,31,32^.﻿ Furthermore, to realize ambitious emissions reductions, FSs actors must coordinate and promote improvements on several other fronts, including institutional capacity (i.e., governance), finance, research, and technical assistance, especially in developing and least developed countries, and plan major emission reductions in the short run using current cost-effective practices. This would improve the feasibility of net-zero commitments and make FSs less dependent on the success and affordability of new-horizon technologies for large-scale negative emissions (which are uncertain at the moment) and cause carbon-intensive industries to stop growth and move to less intensive options.

The mitigation potential of FSs interventions must also be validated against efficacy and cost-effectiveness across regions to avoid unintended consequences and minimize trade-offs^39,40^, which safeguards the effectiveness of practices in reducing emissions and enhancing food production and security. To support this process, research could be directed to tailor practices for different contexts, while making affordable new-horizon technologies in the medium- and long-term. This process must be done in close coordination with technical assistance for effective adaptation and implementation of mitigation and carbon sequestration practices on the ground along with farmers, in conjunction with assistance to meet monitoring, reporting and verification (MRV) of emissions requirements^41^. Science-based targets (FLAG) could be a reference as well as carbon market standards (e.g., VERRA and Gold Standard). Global benchmarks^11^ must also be kept up to date to track the implementation of food system actions and commitments.

Critically, the reorientation of both public and private sector sources of capital is needed to achieve net-zero emissions in global food systems by 2050. Firstly, financial mechanisms supporting the adoption of practices to realizing net-zero could be created by orienting traditional bank loans for positive climate impact, and scaling other approaches, such as blended finance and carbon markets^42,43^. Traditional bank loans offer a pathway to scale validated cost-effective technologies given the position of the lender to incentivize technology adoption. However, following the experience in the sector of renewable energy and energy efficiency, this requires access to patient capital and technical assistance for building the capacity of financial intermediaries, especially in developing and least developed countries, to construct loan portfolios and design incentive mechanisms that are explicitly linked to climate outcomes (e.g., Global Climate Partnership Fund—GCPF). The public sector can support in developing institutional frameworks such as cost-effective assessment and monitoring frameworks to enable the growth of such portfolios.

Secondly, inovative financial mechanisms are needed to demonstrate the viability of investments in the adoption of low-emission interventions and carbon sequestration practices in developing and least developed countries, as well as absorb some of the early risk and up-front cost associated with a shift away from business as usual. Strategically allocating public sector capital to de-risk some of the private sector challenges (i.e. blended finance mechanisms etc.) and incentivizing the private sector to create new investment opportunities (i.e. carbon markets etc.,) are critical transition tools to build a diversified portfolio of cost-effective technologies. Furthermore, overlaying and co-designing such mechanisms with large corporations through, for example, implementing customized and collaborative corporate insetting programs within shared supply chains can ensure buy-in while contributing to the net-zero transition.

Lastly, new funding models are required to sustain inflows of high-risk capital to incubate and accelerate new horizon technologies, especially to move technologies from the investment readiness phase to the implementation phase. Public sector can support in creating an enabling environment for such programs, especially in developing and least developing countries where models are less developed.

Evidence shows that countries with better governance have more effective climate policies and could help maintain the integrity of the net-zero target while avoiding unintended consequences due to policy changes^44,45^. Investing in education, especially in regard to gender, is a key predictor of higher levels of governance. Increasing societal awareness of the need to support changes in food systems and consumption patterns is also fundamental for driving transformational change^14^.

To foster this scenario at a global level, FSs net-zero plans could put more emphasis in the short run on a strong coalition of developing and developed nations, which are likely to have a higher capacity, while build capacity in developing and least developed countries, where international cooperation may also help.

Since our analysis is limited to a global overview, the implications of FSs intensification may have different consequences at regional and country scales. Therefore, it is important that further analysis shed more light on the possibility to mix different intensification strategies to optimally meet socio-economic and environmental targets. Furthermore, data validation (e.g., emission factors and food production) is key for refining findings as well as recommendations for food systems stakeholders. This is especially applied to the levels of emission and emissions reductions while enhancing food production efficiency^16^, as well as carbon sequestration in agriculture-based systems^5^.

Although net-zero FSs are achievable, bolder implementation of more efficient production practices is fundamental to feasibly meet both global food production and climate goals. This work provides an overview of this challenge along with a vision that could guide FSs actors towards these objectives.

## Methods

To estimate current and future FSs emissions and design strategies to achieve net-zero emissions by 2050, we evaluated emissions from 19 major crop and livestock (food) value chains by multiplying their respective global domestic production projections under business-as-usual^7^ by a range of value-chain emissions intensities (10th, 20th, 30th, 40th pctl of least emission-intensive systems and average)^16^ (SM). This approach permits to estimate total food value-chain emissions at different emission intensities (percentiles) that can be further used to evaluate potential changes in emissions by shifting production system efficiency. Although there has been a business-as-usual increase in food production efficiency, this rapid assessment assumed that the business-as-usual FS GHG emissions per unit of food produced remain constant at current levels—although we further discuss business-as-usual trends in the main text. For livestock value-chain emissions, we deducted emissions from feed production^16^ to avoid double-counting the emissions from the production of feed ingredients (e.g., grains). Emissions intensities^16^ encompass the emissions of major GHGs released through FS operations from “farm to fork”: carbon dioxide (CO_2_), nitrous oxide (N_2_O), and methane (CH_4_).

We estimated changes in FS emissions starting in 2020 through 2050 for multiple scenarios:

- Implementation of low-emissions interventions to shift production to the 40th, 30th, 20th and 10th pctl of least emission-intensive systems^16^. Lower percentiles are associated with no or reduced land-use change and food loss and waste^15^.
- We considered that this shift would promote carbon sequestration in cropland and grassland soils (through best management practices), above and below-ground agroforestry systems and the application of biochar to soils^5^. We tested the realization of those potentials at 50, 75 and 100%^5^. We did not consider the carbon sequestration potential from afforestation and reforestation (A/R) and other natural ecosystem restoration (e.g., mangroves and peatlands) to FS^5^. We also assumed the eventual spared area used for feed production would be directed to expansion of other crops for human consumption.
- Reduce global production, driven by lower consumption, of livestock-based protein (meat and milk) by 10, 25 and 50%, calculated using the 2050-projected levels as reference^7^. We assumed that reducing consumption of livestock products lowers milk and meat production. This process should slow demand growth, and eventually reduce the number of livestock heads—the major GHG source in the agricultural sector.
- Adoption of new-horizon technologies across the food value-chains. These technologies include those that are not yet present on farms but could increase mitigation from GHG–efficient food production practices, land-use change, and carbon sinks^30^, as well as make current cost-ineffective practices and technologies affordable^5^.

We built a pathway towards 2050 by assuming these strategies would be implemented at a rate of 20, 50 and 100% by 2030, 2040 and 2050, respectively. For livestock and rice production, we adjusted^16^ data to reflect the contribution of CH_4_ emissions to warming potential using the GWP* concept^25–27^. Under GWP*, stable CH_4_ emission rates contribute a relatively small CO_2_e emission. Increasing CH_4_ emission rates are reflected as a large CO_2_e emission and can exceed the GWP_-100_ of CH_4_ if rates increase at more than approximately 1% per year. Declining CH_4_ emission rates are reported as a negative CO_2_e emission and can reach zero CO_2_e if emission rates decline by 0.35% per year over 20 years. For that, we consider 2020 as the base year where the GWP* concept was applied. We must also consider that approximately 70% of the emissions from livestock and rice production are in the form of CH_4_^11^ and that approximately 70% of these emissions come from farm level^16^.

Despite providing 60+ pathways for achieving net-zero FS using a transparent and accessible methodology and framework, certain limitations and gaps remain, especially on data sources related to the FS emissions factors and carbon sequestration potentials used in this work. For example, the development of FS emission factors percentiles relied on several studies evaluating emissions across a number of food value-chains^16^. As some of those studies reported group farms into a single observation and/or provided an impact average and its associated standard deviation, to include intrinsic sources of variance across parts of the value-chain and across observations (e.g., emissions factors, processing, packaging, retail, and transport impacts; processing conversions; and other conversions), the authors re-specified all values associated with variance as normally distributed variables. As pointed out by the authors, this approach may have limitations if studies are not reporting standard deviations or if they are remodeling from inventory data were used to fill different emissions gaps for each study. Nevertheless, the approach was likely one of the best way to incorporate multiple sources of variance found across studies to develop emissions percentiles. Similar limitations may also apply to the carbon sequestration dataset used in this work^5^, it also relies primarily on several previous research to derive carbon sequestration potentials. However, by updating global and regional mitigation potentials using both sectoral and integrated assessment model (IAM) approaches and comparing the results of both approaches, this study significantly improved the estimation of land-based mitigation potentials. Additional research is however needed for validating key datasets for estimating emissions and removals in FS across difference geographies and contexts and, ultimately, refining recommendations for FS stakeholders. This is especially applied to the attainable levels of emission and emissions reductions while enhancing food production efficiency^16^, as well as carbon sequestration in agriculture-based systems^5^ (Supplementary Information).

## Supplementary Information

Supplementary Information.

## Data Availability

Datasets generated and/or analysed during the current study are available in the article supplementary material.
